# Uropathogenic *Escherichia coli* Modulates Immune Responses and Its Curli Fimbriae Interact with the Antimicrobial Peptide LL-37

**DOI:** 10.1371/journal.ppat.1001010

**Published:** 2010-07-22

**Authors:** Ylva Kai-Larsen, Petra Lüthje, Milan Chromek, Verena Peters, Xiaoda Wang, Åsa Holm, Lavinia Kádas, Kjell-Olof Hedlund, Jan Johansson, Matthew R. Chapman, Stefan H. Jacobson, Ute Römling, Birgitta Agerberth, Annelie Brauner

**Affiliations:** 1 Department of Medical Biochemistry and Biophysics, Karolinska Institutet, Stockholm, Sweden; 2 Department of Microbiology, Tumor and Cell Biology, Division of Clinical Microbiology, Karolinska Institutet and Karolinska University Hospital, Stockholm, Sweden; 3 Department of Pediatrics, CLINTEC, Karolinska University Hospital and Karolinska Institutet, Stockholm, Sweden; 4 Department of Microbiology, Tumor and Cell Biology, Stockholm, Sweden; 5 Swedish Institute for Infectious Disease Control, Solna, Sweden; 6 Department of Anatomy, Physiology and Biochemistry, SLU, The Biomedical Centre, Uppsala, Sweden; 7 Department of Molecular, Cellular, and Developmental Biology, University of Michigan, Ann Arbor, Michigan, United States of America; 8 Department of Nephrology, Danderyd University Hospital, Karolinska Institutet, Stockholm, Sweden; University of Edinburgh, United Kingdom

## Abstract

Bacterial growth in multicellular communities, or biofilms, offers many potential advantages over single-cell growth, including resistance to antimicrobial factors. Here we describe the interaction between the biofilm-promoting components curli fimbriae and cellulose of uropathogenic *E. coli* and the endogenous antimicrobial defense in the urinary tract. We also demonstrate the impact of this interplay on the pathogenesis of urinary tract infections. Our results suggest that curli and cellulose exhibit differential and complementary functions. Both of these biofilm components were expressed by a high proportion of clinical *E. coli* isolates. Curli promoted adherence to epithelial cells and resistance against the human antimicrobial peptide LL-37, but also increased the induction of the proinflammatory cytokine IL-8. Cellulose production, on the other hand, reduced immune induction and hence delayed bacterial elimination from the kidneys. Interestingly, LL-37 inhibited curli formation by preventing the polymerization of the major curli subunit, CsgA. Thus, even relatively low concentrations of LL-37 inhibited curli-mediated biofilm formation in vitro. Taken together, our data demonstrate that biofilm components are involved in the pathogenesis of urinary tract infections by *E. coli* and can be a target of local immune defense mechanisms.

## Author Summary

Most infections of the urinary tract are caused by uropathogenic *E. coli*. On abiotic surfaces, these bacteria are able to form biofilms, which protect them from various adverse environmental conditions. In this study, we sought to investigate whether two *E. coli* biofilm components, curli fimbriae and cellulose, provide a similar protection against innate immune defense mechanisms of the urinary tract. We put special emphasis on the interaction with the human antimicrobial peptide LL-37, which plays a crucial role in the protection against uropathogenic *E. coli*. We demonstrate that curli expression specifically reduces bacterial sensitivity to LL-37 by binding the peptide before reaching the bacterial cell membrane and exhibiting its bactericidal activity. A more general protection is mediated by cellulose, possibly by hiding immunogenic surface structures of the bacterium. In addition to providing protection, curli are also targeted by the immune system. The formation of new curli fibers is inhibited in the presence of LL-37. Moreover, curliated bacteria show higher immunogenicity than their non-curliated counterparts. Cellulose expression, on the other hand, appears to impair initial host colonization. In conclusion, our findings demonstrate an example of the tight interplay between bacterial virulence factors and the host immune defense.

## Introduction

It has been recognized that bacteria in their natural milieu seldom grow as non-differentiated, single cell organisms. Instead, they form multicellular communities, biofilms, showing coordinated behavior [1]. Classically, biofilm formation includes surface adherence, cell-cell interactions, and production of extracellular matrix [2]. The extracellular matrix contributes to the development of higher-ordered three-dimensional structures that offer advantages to the bacteria, such as increased resistance to antimicrobial substances, mechanical forces and to nutrient depletion [3]–[5]. During urinary tract infections (UTI), the role of bacterial biofilms has previously been established in the presence of indwelling catheters [6]. However, uropathogenic *E. coli* also forms biofilm-like structures on and inside host cells in the absence of a foreign body [7]–[9], and the ability to form biofilms has been related to persistence of bacteria in the urinary tract [10].

Curli belong to a class of fibers known as amyloids [11] and are involved in adhesion to surfaces, cell aggregation and, finally, biofilm development. Functionally and genetically, curli are linked to cellulose [12], another extracellular matrix component of biofilms formed by bacteria from the family Enterobacteriaceae. Bacterial cellulose has mostly been investigated in soil bacteria of the family Rhizobiaceae, where this polysaccharide is required for the firm adherence and aggregation of bacteria at the root hair tip of plants [13]. Although the production of cellulose is common among many bacterial species, its biological function and role during infection is not entirely clear. When cellulose is expressed together with curli, the two substances produce a highly inert, hydrophobic extracellular matrix around the bacteria [14]. Biofilms built from curli and cellulose have widely been investigated on abiotic surfaces [15], [16] and in commensal intestinal *E. coli* isolates [17], [18]. Less information is available about the role of curli and cellulose during *E. coli* infection of the urinary tract [10], [19].

Recently, we demonstrated that epithelial cells of the urinary tract up-regulate the production of the human antimicrobial peptide LL-37 upon infection with uropathogenic *E. coli* [20]. Thus, the cathelicidin LL-37 plays an important role in the protection against infections of the urinary tract. The proform of LL-37, hCAP-18, is mainly produced by epithelial cells and neutrophils [21], [22]. After processing [23], the active LL-37 peptide is released and exhibits its bactericidal activity by interaction with the bacterial cell membrane [24].

In the current project, we sought to study the presence of curli and cellulose in *E. coli* isolated from uncomplicated community-acquired UTI and their impact on early UTI pathogenesis. In addition, we here investigate the influence of LL-37 on curli-mediated biofilm formation in *E. coli*. We suggest that curli and cellulose protect the bacterium from immune defense mechanisms and in addition modulate the immune response of the host. We furthermore demonstrate an interaction of curli and LL-37, especially that LL-37 inhibits the polymerization of CsgA, the major subunit of curli.

## Results

### Uropathogenic *E. coli* isolates have higher adhesion capacity and produce more biofilm than commensal *E. coli*

**Figure 1:**
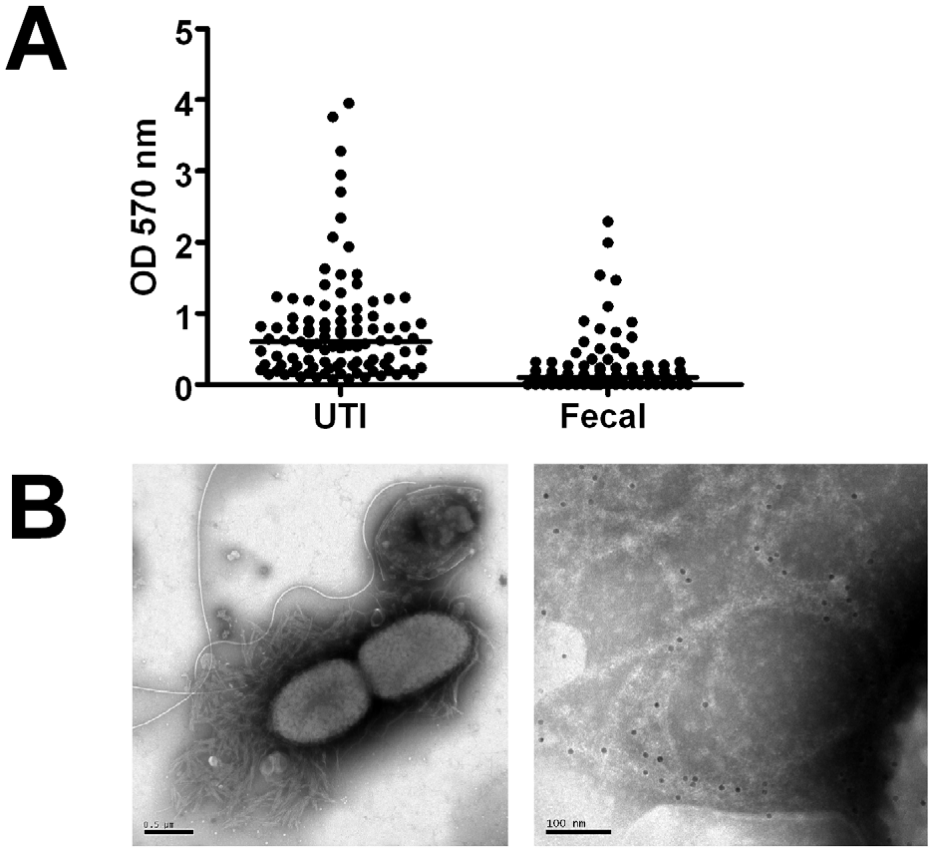
Biofilm expression by uropathogenic and fecal *E. coli* isolates. (**A**) Adhesion capacity and thickness of biofilm produced by *E. coli* isolates collected from urine of patients with urinary tract infections (UTI, *n* = 99) and from fecal samples of healthy individuals (Fecal, *n* = 77) was measured. Individual values and medians are presented, depicted as optical density (OD) at 570 nm after dissolution of crystal violet. The difference is significant (*P*<0.0001, Mann-Whitney U test). (**B**) Isolates from urine samples were investigated by electron microscopy. The left image shows an overview, the right image is a magnification showing immunogold-labelled curli. The scale bars show 0.5 µm (left) and 100 nm (right), respectively.

A total of 99 *E. coli* isolates were collected from urine of patients with UTI and 77 isolates were obtained from fecal samples of healthy individuals. Each isolate was assessed for biofilm formation using a standard microtiter assay (see Materials and Methods). On average, uropathogenic bacteria adhered significantly better and formed more biofilm as compared to fecal isolates (*P*<0.0001, Figure 1A).

### Uropathogenic and commensal *E. coli* isolates produce extracellular matrixes with similar composition

Curli and cellulose production by all isolates was monitored on Congo red and Calcofluor containing plates. To better mimic the host environment, we chose to analyze bacteria grown at 37°C. Based on the uptake of Congo red and fluorescence after the exposure of Calcofluor plates to UV light, we could identify that approximately half of the uropathogenic and commensal *E. coli* isolates expressed curli (54% and 45%, respectively), 30% of the uropathogens and 16% of the commensals expressed curli and cellulose together. This morphotype was significantly associated to uropathogenic *E. coli* (*P* = 0.032). The expression of cellulose alone was rarely detected in either collection (5% and 10%, respectively). Nearly all isolates were positive for expression of type 1 fimbriae, irrespective of their origin (99% of the uropathogenic and 92% of the fecal isolates).

### Curli are expressed in isolates from fresh urine samples

To confirm the expression of curli in vivo, we collected fresh urine samples from patients with community-acquired *E. coli* UTI. Bacteria were analyzed directly from the urine by dot blot analysis and electron microscopy. Ten of seventeen investigated clinical *E. coli* isolates (59%) reacted with antibodies towards CsgA. This was in line with their Congo red/Calcofluor phenotype and the overall prevalence of curli in uropathogenic *E. coli* investigated here (54%). Bundles of curli expressed during UTI were visualized by electron microscopy and their identity was confirmed by gold-labeled antibodies (Figure 1B).

### Expression of curli increases the propensity of *E. coli* to cause infection

**Figure 2:**
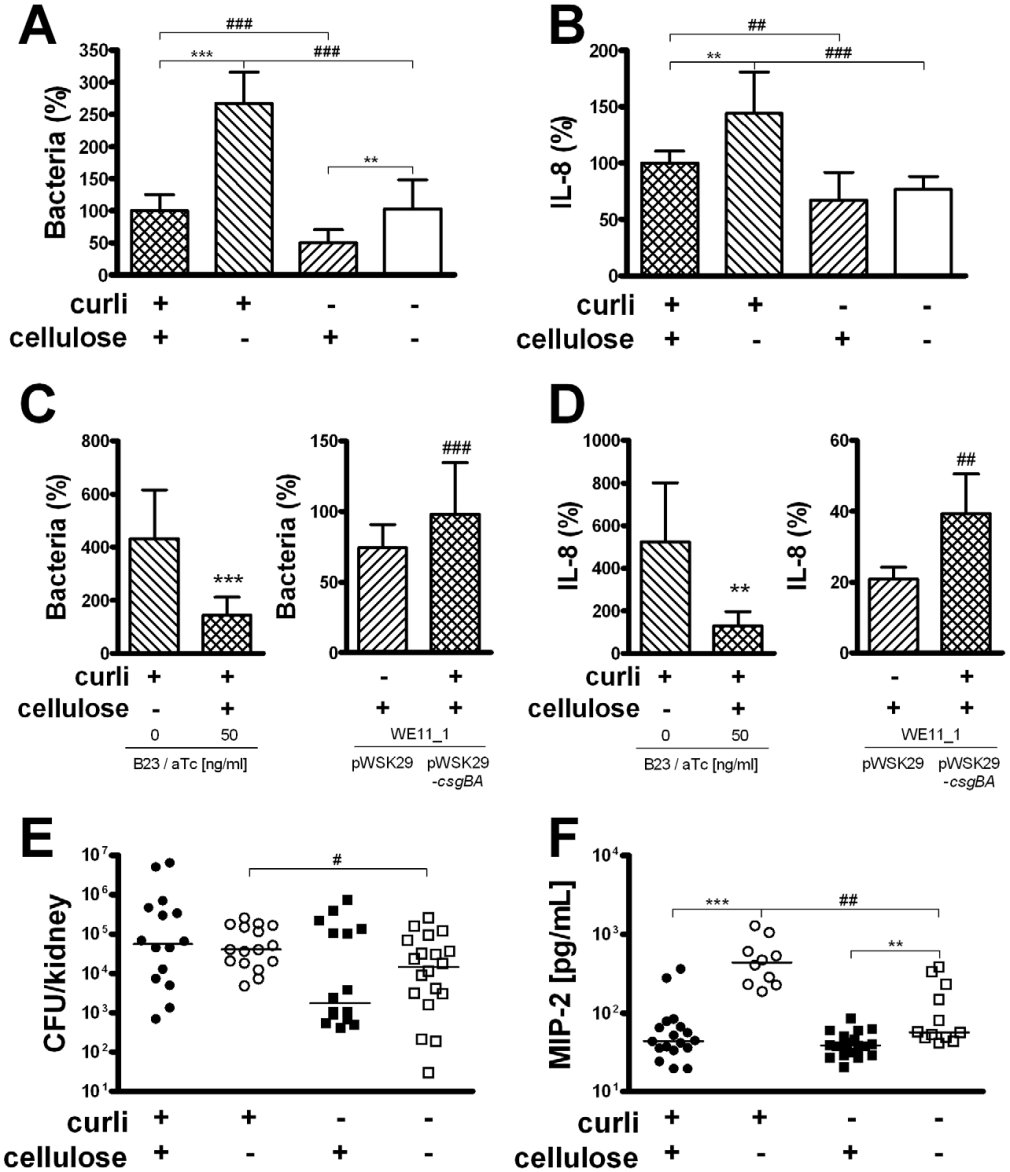
Adhesion and immune induction by *E. coli* expressing or lacking curli and/or cellulose. (**A**) Adhesion to renal epithelial cells A498 was measured after 30 min. Curliated strains adhered significantly better to renal epithelial cells than strains lacking curli, independent of the expression of cellulose (^###^ *P*<0.0001, *t*-test). Cellulose expression decreased the number of cell-associated bacteria in curliated (^***^ *P*<0.0001, *t*-test) and non-curliated strains (^**^ *P* = 0.001, *t*-test). Results from three independent experiments in quadruplicates are shown as mean and standard deviation. Similar results were obtained for bladder epithelial cells (data not shown). (**B**) Induction of IL-8 was measured in culture supernatants of renal epithelial cells A498 stimulated with *E. coli* for 24 h. Curliated bacteria induced a significantly stronger IL-8 response than the mutants lacking curli in the presence (^##^ *P* = 0.001, *t*-test) and absence (^###^ *P*<0.0001, *t*-test) of cellulose. In curliated bacteria, the expression of cellulose reduced IL-8 induction (^**^ *P* = 0.001, *t*-test). Results from three independent experiments in quadruplicate are shown as mean and standard deviation. Similar results were obtained for bladder epithelial cells (data not shown). (**C+D**) The phenotype of *E. coli* No. 12 could be restored by complementation of its mutants. Cellulose expression in strain B23 is inducible by aTc (left panels) and reduces adherence and IL-8 induction (^***^ *P*<0.0001 and ^**^ *P* = 0.007, respectively, *t*-test). The curli subunits CsgA and CsgB are expressed from pWSK29-*csgBA* in strain WE11_1 (right panels) and increases adherence and IL-8 induction compared to WE11_1 carrying the vector pWSK29 only (^*^ *P* = 0.048 and ^**^ *P* = 0.003, respectively, *t*-test). Results in A498 cells are shown as mean and standard deviation. Data from three experiments in quadruplicate for adherence and from two experiments in triplicate for IL-8 induction are presented. (**E**) Mice were infected with the isogenic *E. coli* strains for 1 h. The curliated mutant were isolated from kidneys in significantly higher numbers than the double knockout (^#^ *P* = 0.026, Mann-Whitney U test). Individual values from *n* = 8–10 mice/group and medians are shown. (**F**) Levels of MIP-2 were measured in kidney tissue of infected mice after 16 h. In the absence of cellulose, the curliated mutant strain induced higher levels of MIP-2 compared to the non-curliated strain (^##^ *P* = 0.001, Mann-Whitney U test). Expression of cellulose reduced the induction of MIP-2 in the presence (^***^ *P*<0.0001, Mann-Whitney U test) and absence of curli (^**^ *P* = 0.001, Mann-Whitney U test). Individual values from *n* = 5–10 mice/group and medians are shown.

The relevance of curli and cellulose expression on bacterial adhesion and immune induction in target cells was investigated by the interaction of bacteria with human cells in vitro (Figure 2). Bladder (UROtsa, T24) and renal (A498) epithelial cells were infected with the uropathogenic *E. coli* strain No. 12, producing curli and cellulose; and its isogenic mutants lacking curli and/or cellulose. The wild-type strain and its mutants also expressed type 1 fimbriae to similar extent. The total number of bacteria after 30 min of cell infection was determined. Curli expression resulted in an increased number of cell-associated bacteria in the presence or absence of cellulose (*P*<0.0001, Figure 2A). Likewise, levels of IL-8 were significantly higher in supernatants of cells infected with curliated *E. coli* compared to those induced by the respective non-curliated strain (*P* = 0.001 and *P*<0.0001 for cellulose-expressing and lacking strains, respectively, Figure 2B). Cellulose, on the other hand, reduced the ability of bacteria to adhere (*P*<0.0001 and *P* = 0.001 in the presence and absence of curli, respectively, Figure 2A). In curliated bacteria, cellulose expression significantly reduced the induction of IL-8 (*P* = 0.001, Figure 2B). The role of curli and cellulose on adherence and IL-8 induction was confirmed by complementation of the curli and cellulose-deficient mutants which restored the wild-type phenotype (Figure 2C+D).

To confirm the role of curli and cellulose during the initial infection steps, mice were infected with the isogenic *E. coli* strains. After 1 h of infection, the expression of curli increased the number of bacteria significantly only in the absence of cellulose (*P* = 0.026, Figure 2E), whereas the comparison between the wild-type strain and the curli-lacking mutant was not significant. However, comparing the pair of curliated strains with the pair of non-curliated mutants, the effect of curli on adherence in vitro was supported (*P* = 0.007). Similar to the cell culture experiments, the expression of curli alone increased the induction of MIP-2 (*P* = 0.001, Figure 2F). Interestingly, the inhibitory effect of cellulose was even more pronounced in vivo, and was also observed in the absence of curli (*P*<0.0001 and *P* = 0.001 in the presence and absence of curli, respectively, Figure 2F). Moreover, none of the strains expressing cellulose induced MIP-2 levels significantly higher than levels in control mice inoculated with sterile PBS (32–52 pg/ml).

### Bacterial cellulose influences neutrophil recruitment and elimination of *E. coli* from the kidney

**Figure 3:**
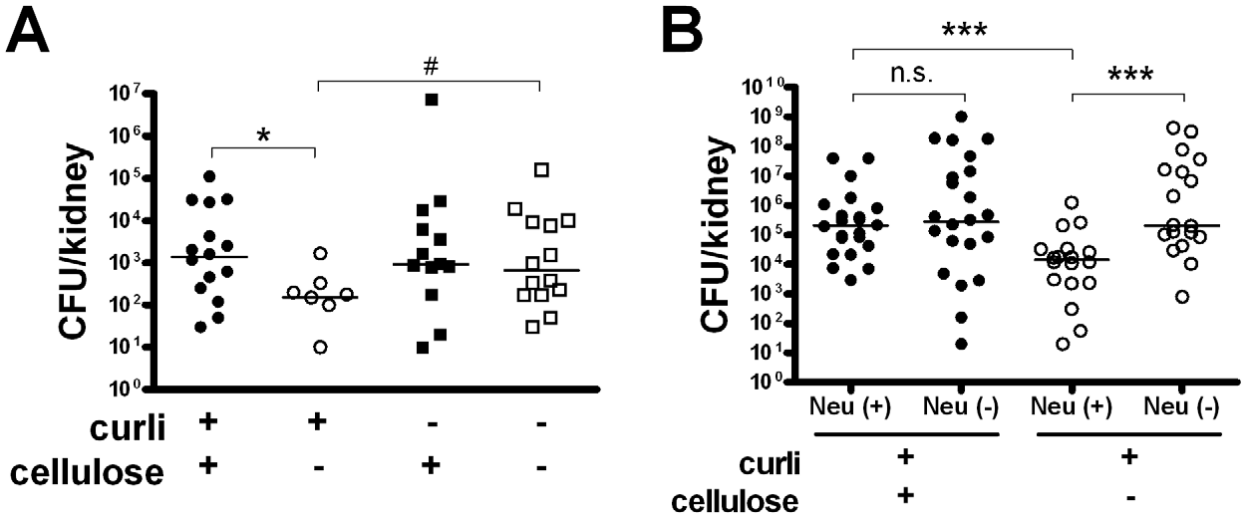
Cellulose delays bacterial elimination in vivo. (**A**) The expression of cellulose in curliated strains increased the number of bacteria in kidneys determined at 48 h p.i. (^*^ *P* = 0.011, Mann-Whitney U test). The expression of curli in the absence of cellulose, on the other hand, mediated a more rapid elimination (^#^ *P* = 0.011, Mann-Whitney U test). Individual values from *n* = 5–8 mice/group and medians are shown. (**B**) Cellulose expression reduces bacterial clearance by neutrophils. Control mice (Neu +) and mice with induced neutropenia (Neu −) were infected with curliated *E. coli* strains, and the bacterial load in the kidneys was determined 48 h p.i. A difference between bacteria with or without cellulose was only seen in the presence of neutrophils (*P* = 0.001, Mann-Whitney U test). Individual values from *n* = 9–12 mice/group and medians are shown.

In the initial stages of UTI, curli promoted colonization (Figure 2A+E). We further investigated the later course of UTI. Mice were infected with isogenic strains expressing curli and/or cellulose, and kidneys were analyzed 48 h post infection (p.i.). MIP-2 is the major neutrophil chemoattractant in the urinary tract [25]. Corresponding to immune induction (Figure 2F), the curliated mutant was more efficiently eliminated after 48 h p.i. than the wild-type strain with cellulose (*P* = 0.011, Figure 3A).

To further investigate the role of cellulose in this process, we induced neutropenia in mice prior to infection. Neutrophil-depleted and control mice were infected with curliated bacteria with or without cellulose. Clearance by neutrophils was more efficient for bacteria lacking cellulose (Figure 3B). In neutrophil-depleted mice, the number of cellulose-deficient bacteria after 48 h was as high as those of the wild-type strain.

### Curli increase resistance to the antimicrobial peptide LL-37

**Figure 4:**
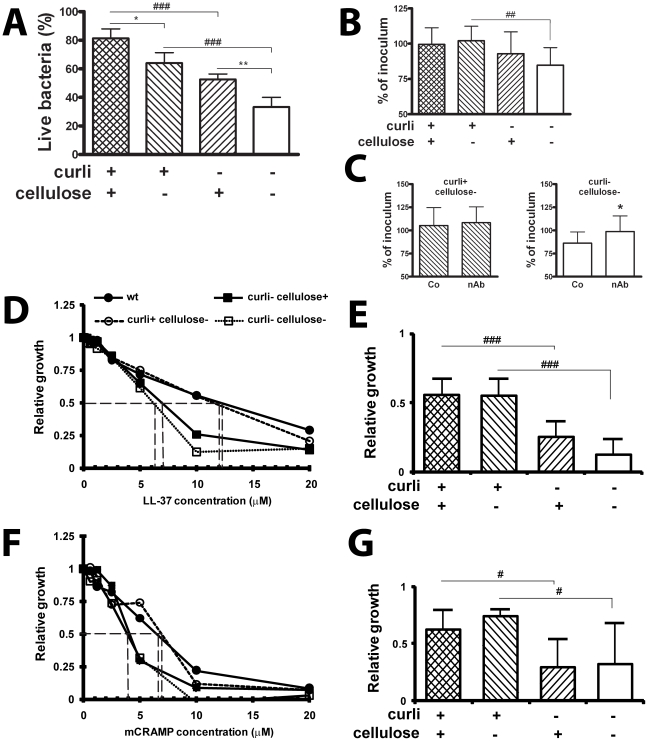
Curli increase the resistance to the antimicrobial peptide LL-37. (**A**) Bladder epithelial cells T24 were infected with bacteria for 30 min and adherent bacteria were subjected to LIVE/DEAD staining. Curli and cellulose expression enhanced bacterial resistance to antimicrobial properties (^###^ *P*<0.001 for curliated strains versus non-curliated strains, ^*^ *P* = 0.023 and ^**^ *P* = 0.003 for cellulose expressing strains with or without curli, *t*-test). Combined data from four experiments are shown. (**B+C**) Bacteria were exposed to conditioned medium of bladder epithelial cells T24 stimulated with phenylbutyrate to enhance LL-37 production. Curli expression enhanced bacterial survival over 30 min (^##^ *P* = 0.006, *t*-test) (**B**). Results from three experiments in triplicate are shown. Conditioned medium was incubated with neutralizing anti-LL-37-antibodies (nAb) or isotype control antibodies (Co) prior to bacterial inoculation. Neutralizing of LL-37 had no effect on viability of the curliated strain (left) but enhanced viability of the double knockout (right, ^*^ *P* = 0.047, *t*-test) (**C**). Results from four experiments in triplicate are shown. (**D–G**) The susceptibility to LL-37 and mCRAMP of *E. coli* strains expressing or lacking curli or cellulose was tested by the broth dilution method. The expression of curli increased the resistance to both LL-37 (**D+E**) and mCRAMP (**F+G**). A significant difference of bacterial growth was observed at 10 µM LL-37 between curliated and non-curliated strains (^###^ *P*<0.001, *t*-test). The curliated strains were also significantly more resistant to 5 µM mCRAMP than bacteria not producing curli (^#^ *P*<0.05, *t*-test). An increased resistance to both cathelicidins was not observed for cellulose. Mean and standard deviation from data of two separate experiments in triplicates are shown. The IC_50_ is indicated by a broken line.

To understand the mechanism underlying the more efficient infection by curliated bacteria, we specifically investigated the antimicrobial activity of bladder and renal epithelial cells on adhered bacteria. For this purpose, bacteria were coincubated with cells for 30 min and adherent bacteria were then subjected to a staining procedure allowing the discrimination between live and dead bacteria. Dependent on the expression of curli and cellulose, 19% to 67% of cell-associated bacteria were killed. Curli but also cellulose protected bacteria from antimicrobial activities of the cells (Figure 4A, *P*<0.001 for curli and *P* = 0.024 and *P* = 0.003 for cellulose, respectively), most efficiently when both structures were expressed together.

Bladder and renal epithelial cells are known to produce cathelicidins in response to *E. coli* infection, in particular LL-37 in humans and mCRAMP in mice, respectively [20]. To relate the observed antimicrobial activity of uroepithelial cells to this peptide, bacteria were exposed to conditioned medium from cells stimulated with phenylbutyrate to enhance LL-37 production [26]. After 30 min of incubation, the number of curli-producing bacteria was almost unchanged (99% and 102% of the inoculated concentration), whereas bacteria lacking curli were reduced to 93% and 85% in the presence or absence of cellulose, respectively (Figure 4B). The most pronounced difference was observed due to curli in the absence of cellulose (102% versus 85%, *P* = 0.006). Hence, we chose these two mutant strains for neutralizing experiments. Prior to inoculation, the activity of LL-37 in the culture medium was inhibited by neutralizing antibodies. While the number of viable curliated bacteria did not differ after 30 min (Figure 4C, left), the number of bacteria lacking curli was significantly higher in the presence of LL-37-specific antibodies compared to the samples treated with an irrelevant isotype control antibody (Figure 4C, right, *P* = 0.047).

We further investigated the influence of curli expression on bacterial sensitivity to LL-37 and mCRAMP more specifically by a broth dilution method. When bacteria were initially grown in biofilm, the concentration of LL-37 at which bacterial growth was inhibited to 50% (IC_50_) was 12 µM for the curliated strains. However, the IC_50_ for the non-curliated strains was only 6–7 µM (Figure 4D). At 10 µM LL-37, the relative growth of the curliated strains was significantly higher than growth of the non-curliated strains (*P* = 0.001 and *P*<0.001 in the presence and absence of cellulose, respectively, Figure 4E). Similar results were obtained for the mouse cathelicidin mCRAMP (Figure 4F+G). The IC_50_ value was higher for the curliated strains than for the non-curliated strains (7 versus 4 µM, Figure 4F). At 5 µM mCRAMP, the relative growth differed significantly between the curliated and the non-curliated strains (*P* = 0.034 and *P* = 0.037 in the presence and absence of cellulose, respectively, Figure 4G). The same bacteria were then pre-grown planktonically, where curli expression is suppressed. When grown under such conditions, no significant difference in the resistance against both LL-37 and mCRAMP was observed between the strains (data not shown). These data indicate that curli is a biofilm component that counteracts the bactericidal effect of cathelicidins and may contribute to the increased resistance of *E. coli* growing in biofilm. In contrast to curli, cellulose did not influence the IC_50_ of cathelicidins (Figure 4D–G).

### LL-37 binds to curli fimbriae

**Figure 5:**
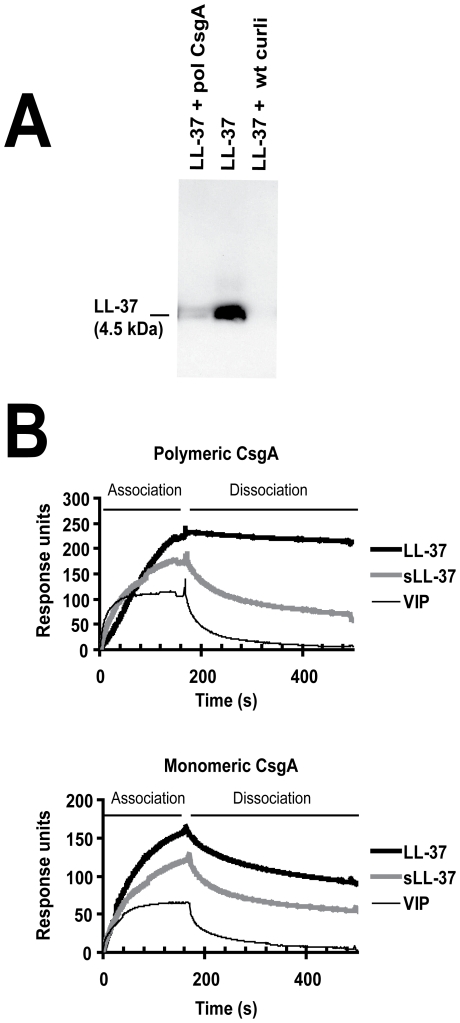
LL-37 binds to recombinant polymerized CsgA and isolated wild-type curli. (**A**) Western blot analysis of supernatants after precipitation of LL-37 with curli. By adding polymeric CsgA (pol CsgA) or wild-type curli (wt curli) to a solution of 0.1 µM LL-37, the levels of LL-37 decreased in the supernatants after centrifugation. (**B**) Surface plasmon resonance. LL-37 exhibits a stronger association and lower dissociation rates to both polymeric (upper panel) and monomeric CsgA (lower panel) compared to the control peptides sLL-37 and VIP.

In order to elucidate one possible mechanism that could influence the increased resistance of curliated bacteria against LL-37, the binding of LL-37 to wild-type curli and recombinant CsgA was assessed. A precipitation assay showed a pronounced decrease of LL-37 in supernatants from samples containing wild-type curli or polymerized CsgA (Figure 5A). Further, LL-37 binding to both monomeric and polymeric CsgA was demonstrated by surface plasmon resonance (Figure 5B). By comparing the response during loading of the peptide in a time frame of 0–180 s, the sensogram of LL-37 demonstrated a higher association with CsgA than the control peptides, i.e. scrambled LL-37 (sLL-37) and the vasoactive intestinal peptide (VIP) [27]. Furthermore, the binding curves reveal that the control peptides had faster dissociation rates than LL-37, indicating a weaker binding to CsgA. This was especially pronounced for the binding to polymeric CsgA. Determination of binding constants was precluded, since LL-37 and in particular CsgA forms oligomers and polymers, respectively, and thus generate several different assemblies.

### LL-37 prevents adherence and biofilm formation in vitro

**Figure 6:**
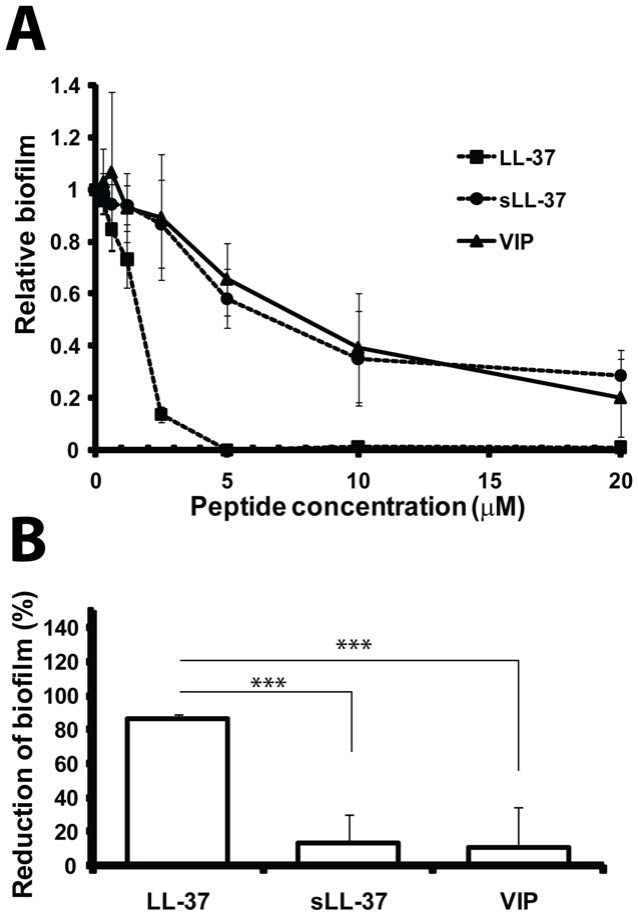
LL-37 prevents formation of biofilm by *E. coli* in vitro. (**A**) Different concentrations of LL-37 and the control peptides sLL-37 and VIP were added to the curli-expressing mutant. (**B**) At 2.5 µM, LL-37 caused more than 80% reduction of biofilm production, whereas the same concentration of the control peptides gave a reduction of only ∼10%. Mean and standard deviation from data of two separate experiments in triplicates are shown. The difference between LL-37 versus sLL-37 or VIP at 2.5 µM was statistically significant (^***^ *P* = 0.001, *t*-test). Similar results were obtained for the wild-type strain expressing both curli and cellulose (data not shown).

At concentrations below the IC_50_ for bacterial growth, LL-37 inhibited curli-mediated biofilm formation with a reduction of more than 80% at 2.5 µM for both the wild-type (data not shown) and the cellulose-negative *E. coli* strain (Figure 6). To investigate the specificity for the inhibitory capacity, sLL-37 and VIP were analyzed in the same assay. Our results showed that the same concentration of these peptides reduced biofilm formation by only 10%, which is a significantly lower reduction than the effect of LL-37 (*P* = 0.001, Figure 6B). This indicates a sequence-specific inhibition of curli-mediated biofilm by LL-37.

### LL-37 inhibits the polymerization of CsgA

**Figure 7:**
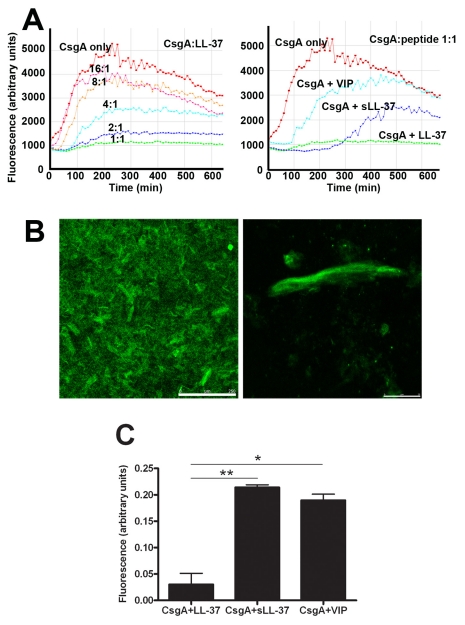
LL-37 inhibits CsgA polymerization. (**A**) Monomeric CsgA was incubated without or with different concentrations of LL-37 (left). The CsgA monomers formed fibers that could be detected by the fiber-specific fluorescent dye Thioflavin (ThT). When bound to fibers, ThT gave rise to fluorescence that was detected by a Tecan plate reader. The fiber formation was inhibited by LL-37 in a dose-dependent manner and was completely inhibited at a molar ratio of 1∶1 (CsgA∶LL-37). As control peptides, VIP and sLL-37 were included (right). (**B+C**) Confocal images of polymerized CsgA stained with ThT. (**B**) The left image shows an overview, the right image a magnification of an aggregate of fibers. Scale bars are 250 µm (left) and 25 µm (right), respectively. (**C**) Fluorescent signals from CsgA incubated with LL-37, sLL-37, or VIP were quantified. Values were corrected for the contribution of the peptides themselves and are expressed in relation to the signal from CsgA alone. The inhibitory effect of LL-37 is significantly stronger than the effect of sLL-37 (^**^ *P* = 0.007) or VIP (^*^ *P* = 0.011). Combined results from two experiments are presented.

To explain a possible cause for the inhibition of biofilm formation by LL-37, the effect of LL-37 on curli formation was investigated. For this purpose, we utilized monomeric recombinant CsgA, the major subunit of curli, which spontaneously polymerizes [28]. CsgA polymerization was monitored with thioflavin T (ThT), a fluorescent dye that binds to polymerized, but not to monomeric CsgA. Our results demonstrated that CsgA polymerization started immediately after incubation at 37°C and reached a stationary phase after approximately 300 min (red line in Figure 7A). After prolonged incubation, the fluorescence declined, most likely due to degradation of ThT and/or precipitation of fibers [28]. The polymerization was inhibited by LL-37 in a dose-dependent manner, and at a molar ratio of 1∶1 (CsgA∶LL-37) fiber formation was completely inhibited (Figure 7A, left panel). The control peptides sLL-37 and VIP had a less pronounced effect on CsgA polymerization than LL-37 (Figure 7A, right panel).

Similar results could be achieved using confocal microscopy. After 20 h incubation of CsgA alone, we could clearly detect fiber structures stained with ThT (Figure 7B) or Congo red (data not shown). In line with the results described above, these fibers were not detected when LL-37 was present in a molar ratio of 1∶1, giving a fluorescence of only 0.05 arbitrary units compared to CsgA alone (1 arbitrary unit, Figure 7C). The control peptides sLL-37 and VIP reduced the fluorescence intensity to approximately 0.2 arbitrary units, suggesting a lower inhibitory capacity than LL-37. Thus, inhibition of CsgA fiber formation was evidently stronger for LL-37 (*P* = 0.007 and *P* = 0.011 versus sLL-37 and VIP, respectively, Figure 7C).

### The structure and the levels of CsgA monomers remain stable in the presence of LL-37

**Figure 8:**
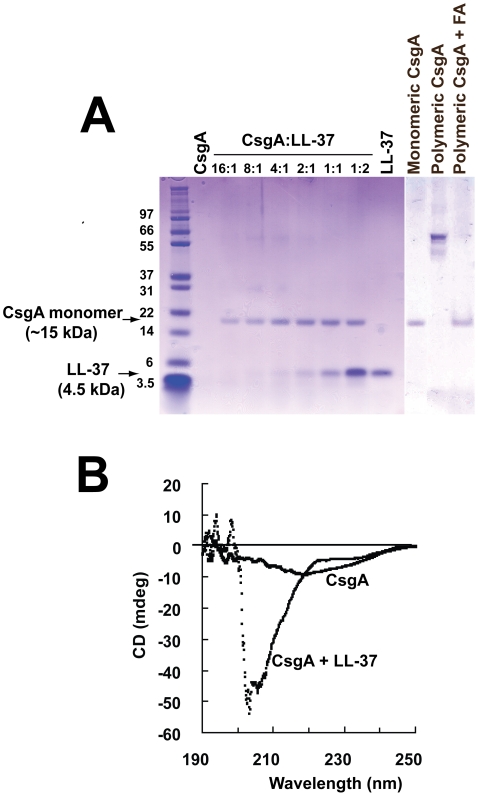
The monomeric form of CsgA remains stable in the presence of LL-37. (**A**) Monomeric CsgA was incubated for 20 h at 37°C without or with LL-37. When CsgA is incubated together with LL-37, the CsgA monomer is visible after SDS-PAGE, whereas the monomer is not detected in the absence of LL-37. When polymeric CsgA is treated with formic acid (FA), the CsgA monomer is detectable, excluding degradation of CsgA. The bands of ∼30 and ∼60 kDa are most likely the dimer and tetramer of CsgA, respectively. (**B**) The stability of the monomeric form of CsgA in the presence of LL-37 was also confirmed with CD spectroscopy after 60 h incubation. The CD spectrum reveals that CsgA alone exhibits a fiber-like structure with weak beta-sheet conformation and a decreased solubility. In contrast, CsgA incubated together with LL-37 displays an unstructured, random coil structure. The spectrum of LL-37 alone was subtracted from the spectrum of CsgA + LL-37.

To confirm the inhibition of CsgA polymerization we sought to analyse the stability of the CsgA monomer in the absence and presence of LL-37. Freshly purified, monomeric CsgA (10 µM) was incubated for 20 h at 37°C without or with different concentrations of LL-37 and was subsequently separated by SDS-PAGE. After staining with Coomassie Blue, bands corresponding to LL-37 and/or CsgA in monomeric, dimeric or tetrameric form were visualized. When CsgA was incubated alone, monomers were not visible although sometimes dimers and/or tetramers could be observed. This finding indicates spontaneous formation of CsgA oligomers and/or larger polymers that can not migrate into the gel due to their size. However, in the presence of LL-37, a band migrating at 15 kDa, the predicted size of monomeric CsgA, could be observed (Figure 8A). This was already seen at a molar ratio of 16∶1 (CsgA∶LL-37). To exclude degradation of CsgA as a possible explanation for the lack of the gel band, polymerized CsgA was treated with 90% formic acid, dissolving polymeric CsgA into monomers. After this treatment, a band corresponding to monomeric CsgA was detectable in the gel (Figure 8A).

The impact of LL-37 on the structure of curli was investigated with circular dichroism (CD) spectroscopy. In line with previous findings, polymeric CsgA exhibited a beta-sheet conformation with a minimum around 218–220 nm (Figure 8B) [28]. Furthermore, the low signal amplitude indicates a decreased solubility due to polymerization (Figure 8B). In contrast, CsgA together with LL-37 displayed a random coil structure as has been described for monomeric CsgA [28]. This result indicates that LL-37 is able to stabilize CsgA in an unstructured form.

## Discussion

In the present study, we show that the majority of uropathogenic *E. coli* from uncomplicated community-acquired UTI adheres stronger and produces more biofilm compared to commensal bacteria. Two major extracellular components in *E. coli* biofilm are curli and cellulose. We here sought to explore their role in the course of UTI and their interaction with the human antimicrobial peptide LL-37. During early stages of UTI, curli promote colonization and immune induction. Cellulose in contrast reduces MIP-2 induction, followed by impaired bacterial eradication by neutrophils. The antimicrobial peptide LL-37 produced by uroepithelial cells and neutrophils in the urinary tract interacts with curli-mediated biofilms. Curli bind LL-37, and thus protects the bacterial cell against the bactericidal activity of LL-37. On the other hand, by binding to CsgA monomers and likely also shorter oligomers, LL-37 inhibits CsgA polymerization and curli formation.

We here for the first time provide evidence that curli are present on *E. coli* in fresh urine of infected patients that are not catheterized (Figure 1B). The expression of curli or cellulose was equally common among *E. coli* isolates from UTI and commensal fecal isolates. However, the combined expression of curli and cellulose was the most common phenotype among uropathogenic isolates whereas most of the fecal isolates expressed only curli.

In the pathogenesis of infection, curli fimbriae have previously been implicated in the attachment and invasion of host cells, interaction with host proteins and activation of the immune system [11], [29], [30]. Cytokine induction by *Salmonella* has been associated with binding of CsgA to toll-like receptor 2 [31], which is expressed on bladder and renal epithelial cells [32]. In our clinical samples, urinary IL-8 levels did not correspond to curli expression, and did not differ between isolates expressing different biofilm morphotypes (data not shown). However, virtually all tested clinical UTI isolates expressed type 1 fimbriae. Since type 1 fimbriae and other bacterial factors such as lipopolysaccharides are potent inducers of IL-8, the impact of curli on IL-8 induction was possibly masked [32], [33]. In addition, it can not be ruled out that the lag time between onset of symptoms and the first visit to the hospital, when urine samples were obtained, also influenced the results.

However, we did observe a clear correlation between curli expression and IL-8 induction in bladder and renal epithelial cells (Figure 2B) as well as MIP-2 in mice infected with the isogenic strains (Figure 2F). Interestingly, curli-dependent IL-8 induction was also observed in A498 kidney cells, which have been found to lack toll-like receptor 2 [32], [34]. It has been reported for this cell line, that IL-8 induction is probably increased by type 1 fimbriae-mediated attachment [32], [35]. We speculate that adherence enhanced by curli could similarly increase the immune induction in A498 cells in our experiments. Another explanation would be that there is an alternative route not yet identified mediating the immune response.

Recruitment of neutrophils is mediated by IL-8 and MIP-2 in humans and mice, respectively [33], [36]. The crucial function of MIP-2 and neutrophils in the defense of the urinary tract [25] is illustrated here by less efficient elimination of the curliated, highly immunogenic cellulose mutant strain in neutrophil-depleted mice (Figure 3B). In contrast, clearance of the wild-type strain expressing cellulose is not significantly influenced by the lack of neutrophils (Figure 3B), consistent with low levels of MIP-2 detected in wild-type infected mice (Figure 2F). It is well known that in wound healing bacterial cellulose itself does not induce inflammation [37]. The role of bacterial cellulose in the pathogenesis of infections, however, has previously not been established. Our results suggest a protective role against the immune system. Cellulose might mask bacterial surface structures, hence avoiding immune recognition and cytokine induction, or alternatively, actively decrease the immune response. In the cell culture model, we see a significant reduction of IL-8 after infection with the wild-type strain compared to the mutant expressing only curli, whereas there is no reduction due to cellulose in the absence of curli. It is reasonable to believe that cellulose might be able to cover the relatively short curli fibers but not longer structures such as type 1 fimbriae, which are expressed by all four strains utilized in this study. In the mouse model, we see a reduced MIP-2 induction in the presence of cellulose also in the absence of curli (Figure 2F). Moreover, the inhibitory effect of cellulose on MIP-2 induction appears to be stronger. Thus, the production of cellulose might be an efficient protection for bacteria not only against environmental conditions but also against immune defense mechanisms in vivo.

We have recently shown that LL-37 plays a crucial role in urinary tract innate immune defense [20]. Here we demonstrate increased resistance of curliated bacteria towards the antimicrobial properties of uroepithelial cells (Figure 4A–C), which is at least partly based on increased resistance against LL-37 (Figure 4C–E). We confirmed the relevance of this observation for the mouse infection model by investigating the susceptibility of wild-type and mutant *E. coli* against mCRAMP, the murine LL-37 ortholog. Our results revealed that the curliated strains are more resistant also against the mouse cathelicidin (Figure 4F+G), indicating a similar interaction with curli as demonstrated for LL-37.

Curli fibers are mainly composed of polymerized CsgA. By precipitation of LL-37 in the presence of wild-type curli or recombinant polymeric CsgA, we demonstrate binding between the peptide and the protein (Figure 5A). Antimicrobial peptides including LL-37 kill their target cells by a peptide-bacterial membrane interaction that leads to lysis of the bacterium [24]. Our finding suggests that LL-37 might be trapped in the net of curli covering the bacterial surface. This prevents LL-37 from reaching the bacterial membrane and lysing the cell. In contrast, bacteria without curli lack this protection and are more easily killed during adherence and invasion into the uroepithelium. A similar protective role against cationic antimicrobial peptides has been observed for the biofilm components alginate in *Pseudomonas aeruginosa* [38] and the polysaccharide intercellular adhesion (PIA) in *Staphylococcus epidermidis* [39]. We also observed partial protection mediated by cellulose, the polysaccharide component in *E. coli* biofilm (Figure 4A+B). However, this effect was not as pronounced as the protection by curli and could not be related to LL-37, since cellulose production did not influence bacterial susceptibility to the cathelicidins LL-37 or mCRAMP in vitro (Figure 4D–G).

Interestingly, we demonstrate that LL-37 inhibits the formation of curli-promoted biofilm formation in vitro (Figure 6). We also show that LL-37 prevents CsgA polymerization (Figures 7+8), and speculate that LL-37 inhibits biofilm establishment by direct interference with CsgA assembly. The binding of LL-37 to both monomeric and polymeric CsgA might block reactive surfaces that are crucial for the CsgA-CsgA interaction [40]. There are five segments in the CsgA amino acid sequence that are conserved and share similarity to each other. They are characterized by conserved Ser, Gln and Asn residues [11], [40], but these repeats also contain acidic residues that may contribute to an electrostatic interaction with the cationic peptide LL-37. Based on the general structures of amyloids [41], each of these repeats is predicted to form a strand-loop-strand motif in a strong hydrogen bonding network [11], which might be prohibited by the binding of LL-37. Considering the function of curli during infection, the prevention of curli generation would provide an effective host defense mechanism. Our in vivo results demonstrate, despite the initial advantage of curliated bacteria, that they are eradicated more efficiently at later stages of infection. The expression of many virulence factors is highly regulated by environmental conditions, and this has also been shown for curli. Curli are maximally expressed in stationary phase and participate in the initial stage of biofilm, i.e. irreversible attachment, whereas expression might be down-regulated during biofilm maturation correlating to later stages of infection [11], [42]. Reduced curli expression by bacteria colonizing the kidney makes them more vulnerable towards LL-37. In addition to increased bacterial sensitivity, incoming neutrophils release high amounts of LL-37 that contributes to the antibacterial defense. In growing bacteria, the generation of new curli fibers might be inhibited by LL-37, reducing both protection and ability to colonize the host tissue. Remarkably, biofilm-inhibitory concentrations of LL-37 were much lower than bactericidal concentrations and within a range which can be present in vivo [20], [43]. In contrast, subinhibitory concentrations of exogenous antimicrobial drugs, e.g. aminoglycosides, seem to stimulate bacteria to produce biofilm [44]. Moreover, bacteria grown in biofilm are less susceptible to most exogenous antimicrobial agents [45].

Biofilm inhibition by antimicrobial polypeptides has previously been described for *Pseudomonas aeruginosa*. Both LL-37 [46] and lactoferrin [47] increased bacterial surface motility mediated by type IV pili. A direct interaction with biofilm components was not investigated in these studies. The effect was rather related to an influence of LL-37 on the bacterial gene expression profile [46] or an influence of lactoferrin on free iron [47]. These and our findings stress an important anti-biofilm role of antimicrobial polypeptides in host defense. It is likely that the anti-biofilm activity is a general strategy for these host defense molecules to keep potential pathogenic bacteria more vulnerable to killing in various tissues, including the urinary tract.

In conclusion, we demonstrate that uropathogenic *E. coli* by expressing curli are able to modulate the immune response and display increased virulence. Cellulose, on the other hand, may reduce adherence and immunogenicity by masking bacterial surface structures, thereby evading the immune system. We also show that defense mechanisms in the urinary tract interfere with these biofilm components; curli protect the bacteria from being killed by LL-37, in contrast LL-37 is inhibiting the formation of curli fibers. This inhibition might be an important host defense mechanism in the protection against UTI.

## Materials and Methods

### Patients

The studies have been approved by the ethics committee of the Karolinska University Hospital, and written informed consent has been obtained from the patients and parents of the children, respectively, in accordance with the ethics permission.

The clinical study included 98 patients with UTI; 36 children [20] and 62 adult women [48]. One woman suffered from two episodes of UTI with different *E. coli* isolates. The diagnostic criterion of acute UTI was the presence of ≥10^5^ CFU of *E. coli* per ml of freshly voided urine. Except for bacteriuria, the diagnostic criteria of acute pyelonephritis were: body temperature ≥38°C and laboratory signs of systemic inflammation, either C-reactive protein ≥20 mg/liter or erythrocyte sedimentation rate ≥20 mm/h, respectively. In addition, fecal commensal *E. coli* isolates were collected from 77 adults in connection with routine outpatient health examination. None of them had a history of symptomatic UTI or recent gastrointestinal disease, and their urine did not yield *E. coli* on cultivation [48].

### Human cell lines

Two human bladder epithelial cell lines and one human renal epithelial cell line were utilized. Virus-immortalized bladder epithelial cells UROtsa were kindly provided by Prof. Scott Garrett, Department of Pathology, University of North Dakota and cultured as described previously [20], [49]. Bladder epithelial cells T24 (HTB-4; American Type Culture Collection (ATCC), Rockville, MD, USA) were cultured in McCoy's 5A medium containing glutamine (Invitrogen Life Technologies, Carlsbad, CA, USA) supplemented with 10% FBS (Invitrogen). Human renal epithelial cells A498 (HTB-44; ATCC) were cultured as described before [20].

### Bacteria for in vitro and in vivo experiments

For further investigation, *E. coli* isolate No. 12 from a child with pyelonephritis was chosen. This was a typical isolate expressing both curli and cellulose as well as type 1 fimbriae and yielding an approximately median level of biofilm as measured on microtiter plates. One-step knockout of *bcsA* and *csgBA* was carried out according to the protocol of Datsenko and Wanner with modifications [17], [50]. The following mutants were constructed using oligos listed in Table 1; WE1 *bcsA*::Cm, deficient in cellulose production; WE11 *csgBA*::Cm, deficient in curli production; and WE16 *csgBA bscA*::Cm, deficient in both cellulose and curli production. Expression of curli and cellulose in strain No. 12 was confirmed by these knockouts. Production of type 1 fimbriae under the culture conditions used for experiments was confirmed by yeast agglutination (see below).

**Table 1:** Oligos and plasmids used for mutant construction and complementation.

Plasmids or oligos	Relevant characteristics	Source/reference
Plasmids^a^		
pWSK29	low-copy-number vector for cloning and gene expression in *E. coli*; Amp^R^	[52]
pWSK29-*csgBA*	pWSK29::*csgBA*; *csgBA* amplified from strain No. 12; cloned using *Eco*RI and *Hin*dIII restriction sites	this study
pZEtetR21-gfp	plasmid with the *km*RExTET cassette; Km^R^	[53]
Oligos^b^		
Ec_bcsA_Start	-atgatcctgacccggtggttgcttatcccgccggtcaacg gtgtaggctggagctgcttc-	[62]
Ec_bcsA_TGA	-tcattgttgagccaaagcctgatccgatggttgtgccgtca tatgaatatcctccttagt-	[62]
Ec_csgBA_Start	-atgaaaaacaaattgttatttatgatgttaacaatactgggt gcgcctgggattgc**gtgtaggctggagctgcttc** -	this study
Ec_csgBA_Stop_3	-gcggtcgcgttgttaccaaagccaacctgagtgacgttaa c**catatgaatatcctccttagt** -	this study
Csg fw	-agagagaattcgtttagaaatgatagaaaagttg-	this study
Csg rev	-actaaaagcttcttgcgccctgtttctgtaatac-	this study
*km*RExTET-bcs fw	-gtctcatgaacggtacggttatttcatagggatcaagca aa**actagtgcttggattctc** -	this study
*km*RExTET-bcs rev	-gcctatcgcgggatcaggcagagtatctggttcattgtt attcat**ggtacctttctcctctttaatg** -	this study
bcsE control fw	-ccaaccatgagcgaagccgctcg-	this study
yhjQ control rev	-cacctactggttgcttagccgcc-	this study

a AmpR: resistance to ampicillin; Km^R^: resistance to kanamycin.

b Underlined sequences: restriction enzyme recognition sites; bold font: sequence for amplification of the pKD46 resistance/*km*RExTET cassette.

For relevant control experiments, complementation equivalents for the mutants WE1 and WE11 were constructed. The complementation of strain WE11 was achieved as follows: First, the chloramphenicol cassette of the curli-deficient strain WE11 *csgBA*::Cm was removed by Flp-catalyzed excision as described elsewhere [51]; resulting in the Cm-sensitive, curli synthesis-deficient strain WE11_1. The removal was confirmed by PCR using oligos Csg fw and Csg rev (Table 1). The DNA region comprising the *csgBA* operon was amplified from strain No. 12 using the above mentioned oligos and cloned into vector pWSK29 [52]. Finally, the obtained plasmid pWSK29-*csgBA* was transferred into strain WE11_1. The ability of strain WE11_1 containing pWSK29-*csgBA* to produce curli was demonstrated by morphotype assessment on Congo red plates.

The complementation of the cellulose production-deficient strain WE1 proved to be more complex, since plasmid-based complementation approaches failed. Therefore, strain B23 was constructed which originates from wild-type strain No. 12 and carries an inducible promoter upstream of the *bcs* operon. In short, the previously described *km*RExTET cassette [53] which contains the anhydrotetracycline (aTc) inducible *tetA* promoter was amplified using oligos *km*RExTET-bcs fw and *km*RExTET-bcs rev (Table 1) and inserted upstream of the *bcs* operon using the protocol of Datsenko and Wanner with modifications [50]. Insertion of the *km*RExTET cassette was confirmed by PCR using oligos bcsE control fw and yhjQ control rev (Table 1). In the resulting strain B23, cellulose production became an aTc-dependent occurrence due to the insertion of the *km*RExTET cassette as previously communicated [54]. In the absence of aTc, the morphotype of strain B23 is consistent to the morphotype of strain WE1 *bcsA*::Cm. In presence of aTc, cellulose production in strain B23 is restored to comparable levels than in the wild-type strain No. 12 as judge on Congo red plates. Thus strain B23 grown under inducing conditions can be used as a complemented equivalent.

### Biofilm assays

#### Microtiter plate method to measure bacterial adhesion and thickness of biofilm

To measure the ability of the bacteria to adhere and to form biofilm a crystal violet assay in polystyrene microtiter plates (Costar, Corning, NY, USA) was performed [17]. Bacteria were grown in Luria-Bertani (LB) broth without salt for 24 h at 37°C without shaking. Biofilm was then stained with crystal violet (3%). The dye was solubilized with ethanol (95%) and the optical density was measured at 570 nm.

#### Morphotype analysis on Congo red and Calcofluor plates

Bacteria were grown at 37°C for 24 h on Congo red and Calcofluor plates and analyzed as described previously [17]. The mutants generated from strain No. 12 served as controls for the analysis of clinical isolates.

#### Expression of type 1 fimbriae

Expression of type 1 fimbriae was tested by mannose-sensitive agglutination of yeast cells. Bacteria were grown in LB broth without shaking to induce expression of type 1 fimbriae, centrifuged and suspended in PBS (approximately 10^10^ CFU/ml). Bacteria were mixed 1∶1 with a suspension of Baker's yeast (*Saccharomyces cerevisiae*, 3% in PBS) and inspected for agglutination. Specificity of the reaction was tested by the inhibitory effect of mannose (5% in PBS). Bacteria were subcultured in broth up to three times before considered negative for expression of type 1 fimbriae. To quantify fimbrial expression in the isogenic strains, the bacterial suspension was subjected to serial two-fold dilution and mixed with an equal volume of yeast suspension. Agglutination was monitored and the optical density of the highest dilution giving a positive result was recorded. Specificity of agglutination was confirmed by mannose sensitivity.

### Cell experiments

All assays were performed using both bladder (UROtsa, T24) and renal epithelial (A498) cells grown on 24-well plates (Costar). Experiments were performed in quadruplicates and repeated at least three times independently. Wild-type and mutant *E. coli* strains were cultured for 24 h at 37°C on LB agar plates without salt to promote the formation of biofilm. Medium was supplemented with ampicillin (100 µg/ml) or aTc (50 ng/ml) if appropriate. Colonies were scraped off and suspended in PBS. Bacterial cell clusters were then removed by centrifugation at 150×*g* for 10 min. The number of bacteria was determined spectrophotometrically at 600 nm and confirmed by viable count on blood agar plates after serial dilutions in PBS.

#### Cell infection

The experiments were performed in serum-free medium supplemented with gentamicin (40 µg/ml). Confluent layers of cells were infected with 10^6^ CFU/ml of *E. coli*. Cells incubated with medium only served as controls. After incubation at 37°C in 5% CO_2_ and 80% humidity for 24 h, medium was aspirated, centrifuged at 350×*g* for 10 min and stored at −20°C prior to ELISA analysis. The viability of cells during the experiments was confirmed using the Trypan blue method.

#### Adhesion assay

To evaluate the ability of bacteria to adhere to epithelial cells, 10^6^ CFU/ml of *E. coli* was added to cell culture wells in serum- and antibiotic-free medium and incubated at 37°C. After 10 or 30 min, cells were washed three times with PBS (37°C) to remove non-adherent bacteria. To collect cell-associated bacteria, ice-cold PBS with 1% Triton X-100 was added. Lysates were plated on blood agar plates after serial dilution in PBS and bacterial numbers were counted after over-night incubation at 37°C.

### Epithelial cell antimicrobial assays

To access the inhibitory activity of epithelial cells on *E. coli* growth and viability two experimental settings were employed.

#### Antimicrobial activity on adherent bacteria

The viability of cell-adherent bacteria expressing or lacking curli and/or cellulose was investigated by LIVE/DEAD staining. For this purpose, *E. coli* were cultured as described above and 10^7^ CFU/ml in serum- and antibiotic-free medium were added to confluent cell layers grown on sterile glass cover slips. After 30 min of incubation at 37°C, non-adherent bacteria were removed by washing three times with PBS. In order to include intracellular bacteria in the staining process, cells were permeabilized with saponin (0.2% in PBS, Sigma-Aldrich, St. Louis, MO, USA) for 5 min before addition of the two-component LIVE/DEAD *Bac*Light stain (Invitrogen) diluted in 0.2% saponin. After 5 min, cells were washed with PBS and lightly fixed in freshly diluted 0.1% paraformaldehyde (PFA) for 15 min. At this concentration, PFA did not affect the fluorescence intensity of any of the dye components. Cover slips were mounted in ProLong Gold antifade mounting medium (Invitrogen) and immediately examined in a Leica TCS SP5 confocal microscope. To ensure correct discrimination between live and dead bacteria, stained green and red, respectively, microscope filter acquisition settings were adjusted using preparations of live and/or dead bacteria. Staining of the cell nucleus with both the cell permeable green dye and the cell impermeable red dye served as a positive control for cell permeabilization. Red or green bacteria were manually counted in microscope images of two separate preparations of three or four independent experiments.

#### Antimicrobial activity of conditioned medium

In order to investigate secreted antimicrobial components and to specify the active compound, bacteria were incubated in conditioned medium. To especially investigate the influence of LL-37 on bacterial viability in relation to curli and cellulose expression, production of LL-37 was stimulated with phenylbutyrate prior to medium collection [26]. Cells were grown in complete medium to reach ∼80% confluence and medium was then exchanged to serum-free medium supplemented with 4 mM 4-phenylbutyrate (Tocris Bioscience, Bristol, UK). After additional 48 h, medium was collected and cells were removed by centrifugation at 300×*g* for 10 min. Bacteria grown as described above and suspended in PBS were added to conditioned medium at a final concentration of 10^4^ CFU/ml. Aliquots of 150 µL were transferred to wells of a polypropylene microtiterplate (Costar) and incubated at 37°C for 30 min with shaking. Thereafter, the number of live bacteria in the conditioned medium was determined by viable count and expressed in relation to the concentration in the inoculum.

To relate the antimicrobial effect to LL-37, monoclonal mouse anti-LL-37 antibodies [55] were added to the conditioned medium at a concentration of 1 µg/ml and incubated for 30 min at 37°C prior to inoculation of bacteria. For control purposes, supernatants were equally treated with mouse IgG1κ isotype control antibodies (BD Biosciences, San Diego, CA, USA). The antimicrobial activity was determined as described above.

### Measurement of IL-8 and MIP-2 levels

Before ELISA analysis, urine was centrifuged at 350×*g* for 10 min to remove cells and larger particles. ELISA kits for human IL-8 or mouse MIP-2 were obtained from R&D systems (Abingdon, UK). IL-8 or MIP-2 levels were determined according to the manufacturer's instructions. The lower detection limit for IL-8 and MIP-2 was 31.3 pg/ml and 15.6 pg/ml, respectively. The urinary levels of creatinine were analyzed colorimetrically, and the levels of IL-8 were expressed as IL-8/creatinine ratios.

### Electron microscopy of *E. coli* in fresh urine

Urine was collected from patients with *E. coli* UTI and without having a catheter. A drop of urine was incubated on carbon/Formvar-coated 400-mesh copper grids (GilderGrids, Lincolnshire, UK) for one minute. Immunostaining was performed as described previously with minor modification [56]. Briefly, grids were blocked with 1% BSA/PBS for 5 min, then incubated with anti-CsgA [28] (1∶200 in 0.1% BSA/PBS) for 60 min at 37°C, followed by incubation with anti-rabbit IgG-10-nm gold antibodies (1∶15 in 0.1% BSA/PBS; Sigma-Aldrich) for 30 min at 37°C. Grids were stained with 2% tungstophosphoric acid (Merck, Darmstadt, Germany) at pH 6. Analysis were performed using a FEI Tecnai Spirit electron microscope (Eindhoven, The Netherlands) at 80 kV accelerating voltage.

### Dot blot analysis

Urine was centrifuged at 300×*g* for 10 min to remove cells and larger particles. Bacteria from the supernatant were collected by centrifugation at 3500×*g* for 10 min. A 2-µL aliquot of the pellet suspended in a minimal volume of PBS was transferred to a nitrocellulose membrane (Invitrogen) and air dried for 15 minutes. Immunostaining was performed as described previously [56]. Briefly, membranes were blocked with 1% milk/1% BSA/PBS for 2 h at room temperature, incubated with anti-CsgA [28] (1∶5000 in 1% milk/1% BSA/PBS) for 1 h, followed by incubation with anti-rabbit horse-radish-peroxidase conjugated antibodies (1∶3000 in 1% milk/1% BSA/PBS; Bio-Rad Laboratories, Hercules, CA, USA) for 1 h.

### Animal experiments

Mouse experiments were approved by the Northern Stockholm Animal Ethics Committee and experiments were carried out according to FELASA guidelines and in compliance with the Committee's requirements.

#### Bacterial infection

Female NMRI mice of 8–10 weeks age were caged in groups of 3–5 animals in standard cages. After water deprivation for 4 h, mice were anaesthetized with isoflurane (Forene™; Abbott Scandinavia, Solna, Sweden) and infected transurethrally with 50 µl of a bacterial suspension of 10^9^ CFU/ml, using a soft sterile polyethylene catheter (outer diameter 0.61 mm, inner diameter 0.28 mm; Clay Adams, Becton Dickinson, Franklin Lakes, NJ, USA) with lubricant. Sterile PBS was used for control mice. After 1, 16 or 48 h, mice were sacrificed by cervical dislocation and bladders and kidneys were aseptically removed. Bladders taken 1 h p.i. were cut open and washed in ice-cold PBS three times to remove non-adherent bacteria. The organs were homogenized in 1 ml PBS and serial dilutions of the homogenate were plated on blood agar plates for viable count. For measurement of MIP-2, homogenized samples were centrifuged at 350×*g* for 10 min and the supernatants were stored at −20°C prior to ELISA analysis. A total of 126 animals including controls were infected. Mice without bacterial growth in any of the organs were regarded as non-infected and therefore excluded from further analysis; only MIP-2 values from infected organs were used for statistical analysis.

#### Neutrophil depletion

To induce neutropenia, 100 µg of RB6-8C5 monoclonal antibody (R&D systems) was administered intravenously 24 h before infection [20]. Control animals received an equal volume of sterile saline. Neutropenia at the time of infection and at the end of the experiment was confirmed on Giemsa-stained blood smears.

### Bacterial sensitivity to LL-37 and mCRAMP

The susceptibility of wild-type and mutant *E. coli* strains to synthetic LL-37 and mCRAMP (Innovagen AB, Lund, Sweden) was determined using a broth microdilution method. Briefly, bacteria were grown overnight at 37°C on LB plates with or without salt, inhibiting or promoting the formation of biofilm, respectively. Then, bacteria were suspended in PBS and diluted in LB broth without salt in a concentration of 10^5^ CFU/ml. The bacterial concentration was verified by viable count after serial dilutions in PBS. Bacteria (90 µl suspension) were grown in 96-well plates in the presence of 10 µl aqueous solution of synthetic LL-37 or mCRAMP in final concentrations ranging from 0.6 µM to 20 µM in 2-fold dilutions. After 20 h, bacterial viability was measured colorimetrically by reduction of Alamar blue (BioSource International, Camarillo, CA, USA) for 1 h at 37°C [57]. The IC_50_ was determined as the peptide concentration that gave 50% reduction of the absorbance at 570 nm relative to bacteria grown without peptide.

### Effect of LL-37 on formation of biofilm

Plates were filled with 90 µL bacterial culture of planktonic cells and 10 µL of aqueous solution of LL-37 in the same concentrations as in the susceptibility assay. As control peptides, sLL-37 (Innovagen AB) and VIP [27] with similar structural properties as LL-37 were utilized. The plates were incubated without shaking at 37°C for 20 h. After incubation, the amount of biofilm formed was determined as described above.

### Purification of recombinant CsgA-His_6_

CsgA-His_6_ and CsgG, a lipoprotein that is required for CsgA secretion [58], were overexpressed in LSR12 (C600::*csg*). The purification procedure of recombinant CsgA-His_6_ was performed as previously described with some modifications [59]. The filtrate was incubated with nickel-nitrilotriacetic acid (Ni-NTA) agarose (Invitrogen) for 1 h at 4°C with shaking, centrifuged at 200×*g* for 5 min and transferred to a polyprep chromatography column (Bio-Rad, Hercules, CA, USA). To confirm protein identity of CsgA-His_6_, the isolated protein was prepared and subjected to SDS-PAGE as described below, and thereafter the protein was transferred from the gel to a polyvinylidene fluoride (PVDF) membrane (Invitrogen) at 160 mA for 60 min. The band of 15 kDa, corresponding to the molecular weight of CsgA-His_6_, was excised from the membrane stained with Coomassie Blue and analysed with N-terminal sequence analysis as has been described [60]. Since it has previously been shown that CsgA-His_6_ has the same polymerizing properties as wild-type CsgA [59], we refer to CsgA-His_6_ as CsgA.

### Isolation of wild-type curli

The cellulose-deficient isogenic mutant of *E. coli* No. 12 was used to purify wild-type curli, as has been described [17]. Colonies were harvested and suspended in 0.05 M Tris-buffer by using an omnimixer. Bacterial cell debris was discarded by centrifugation at 8000×*g* for 15 min and curli protein was precipitated by adding 0.1 M MgCl_2_ and 0.15 M NaCl. The aggregated curli were centrifuged at 16000×*g* for 15 min, and the pellet was dissolved in 10 mM TRIS, 1 mM EDTA, pH 7.5 with 2% 3-((3-cholamidopropyl)dimethylammonium)-1-propanesulfonate (CHAPS). After incubation for 45 min at 95°C, the solution was centrifuged at 20000×*g* for 10 min. The pellet containing curli was washed three times with water. Finally, curli were suspended in PBS and used for binding studies with LL-37.

### Binding of LL-37 to CsgA

#### Precipitation of LL-37 with curli

LL-37 (0.1 µM in PBS) was mixed with 5 µM wild-type curli, or 5 µM recombinant polymerized CsgA. As control, the same concentration of LL-37 without curli or CsgA was utilized. The samples were incubated for 1 h at 37°C with shaking and centrifuged for 30 min at 10000×*g*. An aliquot of the supernatants was analyzed for the presence of LL-37 with Western blot.

#### Surface plasmon resonance

Binding analysis of LL-37 to both monomeric and polymeric CsgA was performed with surface plasmon resonance (Biacore 3000 instrument; Biacore AB, Uppsala, Sweden). Both monomeric and polymeric CsgA was immobilized on a CM5 sensor chip (Biacore AB) surface by amine coupling. The chip surface was normalized in 70% glycerol and lanes on the chip were activated with injection of 35 µL 0.05 M *N*-hydroxysuccinimide (NHS)/0.2 M *N*-ethyl-*N*′-[3-dimethylamino) propyl]carbodiimide (EDC). Polymeric or monomeric CsgA (1 µM in 10 mM sodium acetate, pH 4.5) was then immobilized on the chip to 7000 response units and 2000 response units, respectively. After immobilization, the lanes were subjected to 60 µL ethanolamine (1 M, pH 8.5) to deactivate remaining activated carboxylic groups. One lane, activated and deactivated, was used as negative control (without addition of CsgA). Standard Biacore HBS-EP (Biacore AB) was utilized as running buffer, and 0.1 µM LL-37, sLL-37 or VIP were injected at 20 µl/min for 3 min. The surface was regenerated after each cycle with 100 mM HCl.

### SDS-PAGE

Samples were prepared in LDS (lithium dodecyl sulphate) sample buffer (4∶1) and incubated at 70°C for 10 min. Electrophoresis was performed using 4–12% Bis-tris NU-PAGE gels (Invitrogen) at 200 V for 35 min. Gels were stained with Coomassie Blue for 1 h and destained over night in 90% water, 8% methanol and 2% acetic acid (vol/vol/vol).

### Western blot analysis

Sample preparation and SDS-PAGE was carried out as described above and Western blot was performed as previously described [20]. The antibodies used were monoclonal mouse anti-LL-37 (0.6 µg/ml in 5% fat-free milk/PBS) [55] and horse radish peroxidase-conjugated anti-mouse IgG (diluted 1∶5000).

### CD spectroscopy

Samples containing 40 µM CsgA in 50 mM potassium phosphate buffer (KPi) and 0.02% NaN_3_, pH 7.2, with and without 10 µM LL-37 were incubated for 60 h at 37°C. The samples were then assayed with a Jasco J-810 spectropolarimeter from 190 to 250 nm in a quartz cell with a 1-mm path length at 20°C. The spectrum of buffer alone and LL-37 in buffer was subtracted from the spectra for CsgA alone and for CsgA together with LL-37, respectively.

### Thioflavin T assays

#### Tecan plate reader

Purified recombinant monomeric CsgA (10 µM) with or without different concentrations of the peptides LL-37, sLL-37 or VIP was mixed with 20 µM of the fiber-specific fluorescent probe ThT (Sigma-Aldrich). Fluorescence was measured with a Tecan infinite M200 reader (Tecan Nordic AB, Täby, Sweden). The excitation and emission wavelength was 430 and 490 nm, respectively. Measurements were conducted at 37°C every 10 min after shaking the sample for five seconds. Background fluorescence of the peptides themselves was subtracted.

#### Confocal microscopy

Recombinant monomeric CsgA was mixed with equimolar LL-37, sLL-37 or VIP and 20 µM ThT, and incubated over night at 37°C, washed twice, and suspended in 10 mM Kpi, pH 7.2 before mounting in ProLong Gold antifade mounting medium (Invitrogen). Images were acquired on a Leica TCS SP5 confocal microscope using a 20× objective. Fluorescence intensity was quantified using ImageJ software [61]. The difference in fluorescence intensity of CsgA with and without peptides was calculated and corrected for the contribution of fluorescence from the peptides themselves.

### Data analysis

Data were compared with student's *t*-test, Mann-Whitney U test or Fisher's exact test as appropriate. *P* values of less than 0.05 were considered as statistically significant.
